# Commentary: Psychosocial screening and assessment in oncology and palliative care settings

**DOI:** 10.3389/fpsyg.2015.01223

**Published:** 2015-08-14

**Authors:** Kathrine G. Nissen

**Affiliations:** Department of Psychology, Copenhagen UniversityCopenhagen, Denmark

**Keywords:** screening, attachment theory, attachment style, cancer, psychological distress in cancer

Depression and anxiety are common in patients with cancer and psychosocial screening in oncology and palliative care settings is suggested as a means to reduce emotional distress in cancer situations (Grassi et al., 2014). In their study, Grassi and colleagues report the results of a review investigating factors associated with depression and anxiety in cancer patients. As regards psychosocial screening, the findings are reassuring: assessment of distress enables the practitioner to attend to symptomatology, interpersonal dynamics and cultural aspects of distress, which captures distress as a multifaceted phenomenon. In particular, focusing on the interpersonal aspects, for example attachment, is highlighted as a way to detect distress. There is a long-standing tradition of attachment research within developmental and clinical psychology, but in the field of the psychosocial dimensions of cancer, studies of attachment are of only rather recent of origin. Introducing established attachment measures into health psychology seems, prima facie, a promising avenue. However, important theoretical and conceptual knowledge derived from investigating adult attachment in the contexts of clinical and developmental psychology has been omitted in the transition to the setting of health psychology. Ultimately, this has led to an uncertainty of the fundamental nature of attachment, as is also pointed out by Grassi et al. (2014) in their evaluation of attachment research in the fields of oncology and palliative care. They avoid conceptualizing and defining attachment and important questions therefore remain; to which definition of attachment do the authors adhere? How do they conceptualize attachment? What is the convergence between their definition and the conceptualization? Grassi and colleagues state that “the way in which the patient has experienced early relations with caregiving figures in the past relates to her view of herself and to the expectation (…).” The description of attachment is relevant to the understanding of the phenomenon, and the quotation above indicates that Grassi et al. consider past experiences to be crucial to the attachment construct. They emphasize past relationships in their description of attachment and they make reference to a self-report instrument for measuring attachment, namely the Experience in Close Relationships questionnaire (ECR). Historically, attachment research has split into two schools using two distinct methodological approaches, i.e., the social psychology tradition and the development of parent-child relations from the child psychology tradition (Bouthillier et al., 2002; Ravitz et al., 2010). Attachment measures involving self-report questionnaires such as the ECR (Brennan et al., 1998) and the Relationship Questionnaire (RQ) (Bartholomew and Horowitz, 1991) stem from the social psychological tradition and the aim is to assess adult attachment in the context of current close relationships with a spouse, a relative, or a close friend. Adult attachment, as measured with self-report questionnaires, varies according to the context in which it is measured and it expresses attachment within thoughts and feelings in current close relationships (Brennan et al., 1998). In contrast, adult attachment measured with the Adult Attachment Interview (AAI) originates from the child psychology tradition and it assesses the organization and the processing of attachment experiences in childhood (Hesse, 2008; Ravitz et al., 2010). Given that self-report measurements of attachment and attachment as measured with the AAI are intended to assess the same construct, then the two methodologies could be expected to show at least a moderate association. However, research shows that they fail to correlate (de Haas et al., 1994; Crowell et al., 1999; Bouthillier et al., 2002; Creasey and Ladd, 2005). The distinction between attachment measured with the AAI and attachment as measured with self-reports is important, because, in fact, the two approaches assess two distinct constructs. Therefore, it is necessary to define attachment consistently with the attachment measure being used. Little can be learnt about attachment and psychosocial distress if there is lack of convergence between the measurement instrument employed (e.g., self-report questionnaires) and the description of the phenomenon (e.g., thoughts and feelings in current close relationships or the organization of experiences of child-parent attachment). The evaluation of attachment research in Grassi et al. (2014) is encouraging, but it is also problematic since it omits important distinctions and specifications of the attachment phenomenon. These shortcomings may consequently lead investigators collectively to adhere to an imprecise definition of adult attachment and this in turn will lead to the impact of attachment research within the field of health psychology being considerably diminished. The psychological legacy of attachment research as an approach to improving the health, well-being and rehabilitation of patients and families, needs to continue its development. The challenge for clinicians, service providers and administrative authorities will be to build up the clarity of the phenomenon such that research will be able to establish what attachment is all about.

## Conflict of interest statement

The author declares that the research was conducted in the absence of any commercial or financial relationships that could be construed as a potential conflict of interest.
